# Haralick Texture Analysis for Differentiating Suspicious Prostate Lesions from Normal Tissue in Low-Field MRI

**DOI:** 10.3390/bioengineering12010047

**Published:** 2025-01-09

**Authors:** Dang Bich Thuy Le, Ram Narayanan, Meredith Sadinski, Aleksandar Nacev, Yuling Yan, Srirama S. Venkataraman

**Affiliations:** 1 Promaxo Inc., Oakland, CA 94607, USA; ram@promaxo.com (R.N.); srikalvan@promaxo.com (S.S.V.); 2 Department of Bioengineering, School of Engineering, Santa Clara University, Santa Clara, CA 95050, USA

**Keywords:** feature extraction, Haralick texture, low-field, MRI, prostate cancer

## Abstract

This study evaluates the feasibility of using Haralick texture analysis on low-field, T2-weighted MRI images for detecting prostate cancer, extending current research from high-field MRI to the more accessible and cost-effective low-field MRI. A total of twenty-one patients with biopsy-proven prostate cancer (Gleason score 4+3 or higher) were included. Before transperineal biopsy guided by low-field (58–74mT) MRI, a radiologist annotated suspicious regions of interest (ROIs) on high-field (3T) MRI. Rigid image registration was performed to align corresponding regions on both high- and low-field images, ensuring an accurate propagation of annotations to the co-registered low-field images for texture feature calculations. For each cancerous ROI, a matching ROI of identical size was drawn in a non-suspicious region presumed to be normal tissue. Four Haralick texture features (Energy, Correlation, Contrast, and Homogeneity) were extracted and compared between cancerous and non-suspicious ROIs. Two extraction methods were used: the direct computation of texture measures within the ROIs and a sliding window technique generating texture maps across the prostate from which average values were derived. The results demonstrated statistically significant differences in texture features between cancerous and non-suspicious regions. Specifically, Energy and Homogeneity were elevated (*p*-values: <0.00001–0.004), while Contrast and Correlation were reduced (*p*-values: <0.00001–0.03) in cancerous ROIs. These findings suggest that Haralick texture features are both feasible and informative for differentiating abnormalities, offering promise in assisting prostate cancer detection on low-field MRI.

## 1. Introduction

Prostate cancer is the second most commonly diagnosed cancer and the fourth leading cause of cancer mortality in men [1]. For timely and effective treatment, accurate diagnosis via prostate biopsy is essential. Magnetic resonance imaging (MRI) can be used to radiologically assess suspicious prostate cancer regions prior to biopsy. Offering the urologist a visual estimation of where to sample following the corresponding MRI-suspicious region, transrectal ultrasound (TRUS) biopsy is the current standard of care, yet it has moderate accuracy in the detection of prostate cancer due to blinded sampling [2]. Fusion-guided biopsy, specifically pre-procedure diagnostic MRI fused with TRUS, has been found to be increasing cancer detection rates, reaching fairly accurate sampling of the regions of interest (ROIs). However, deformations of the prostate gland due to the TRUS probe create some variance in the registration of MR images and real-time TRUS images. Also, utilizing intra-procedure imaging as TRUS, in-bore MRI-guided biopsy does not suffer from this problem. In addition, it benefits from the lesion localization capabilities of MRI to improve biopsy guidance in real time but is time consuming, expensive, and not widely available.

More recently, a low-field (<1T) MRI-guided intraoperative approach proposes to address some of these challenges. Low-field MRI is promising for its affordability, compact footprint, and reduced shielding requirements. Low-field MRI offers the benefits of soft tissue contrast, and a similar cancer detection rate compared to in-bore MRI procedures [3]. Allowing MRI-guided transperineal biopsy within a standard urologist’s office, the Promaxo portable, low-field MRI system with an open, single-sided configuration integrates the benefits of MRI-based biopsy for anatomical targeting. This scanner operates at a low, B0 field (58–74mT) with x- and y-axis gradients and a permanent z-gradient. Images are acquired along the transverse direction without endorectal probes with the patient positioned in high lithotomy. During the low-field MRI-guided biopsy, the annotated high-field, T2-weighted MR images are registered to the low-field, T2-weighted images, allowing the urologist to directly target abnormal regions seen on high-field MR images.

Regardless of which image modality is used for guidance, the localization of biopsy targets is still challenging because clinical analysis of MR images for identifying potentially cancerous regions despite global standardization efforts in using the Prostate Imaging–Reporting and Data System (PI-RADS) has been largely qualitative [4]. Quantitative image analysis techniques, such as texture analysis, provide an alternative method that can be more reproducible and less subjective [5]. Texture analysis is a technique to extract frequencies of local spatial variations in signal intensity, thus quantifying pixel relationships within regions of interest. One of the most common image texture analysis techniques is Haralick texture analysis, being studied as quantitative characterization for cancer detection in breast cancer, such as in [6,7,8,9], colon cancer or rectal cancer, such as in [10,11,12,13], or prostate cancer, such as in [14,15,16,17]. Specifically in prostate cancer, Nketiah et al. [14] evaluated four texture features—Homogeneity, Contrast, Correlation, and Entropy—derived from T2-weighted MRI to assess the aggressiveness of prostate cancer, identifying these features as potential diagnostic markers sensitive to pathological variations. Daniel [15] investigated the significance of 20 Haralick texture features extracted from T2-weighted and Apparent Diffusion Coefficient (ADC) images in prostate cancer patients, both with and without neoadjuvant androgen deprivation therapy, with a focus on their potential application in future dose-painting techniques. Shiradkar [17] concluded that Haralick features, derived from pretreatment biparametric MRI, can serve as predictive biomarkers for prostate cancer biochemical recurrence following therapy. In the context of T2-weighted, 3T prostate MRI, Wibmer et al. [18] found that several Haralick-based texture features, including Energy, Contrast, Correlation, and Homogeneity, show promise for prostate cancer detection.

Low-field MRI has distinct differences from high-field images. T2 contrast is impacted by field strength [19]. The signal-to-noise ratio in low-field MRI is inherently lower than that in high-field images [20]. Noise patterns are also different between low-field and high-field MRI. Noise in high-field MR scanners is typically dominated by the object being imaged with additional noise from hardware. At low-fields, object noise is negligible, and overall noise is dominated by hardware components such as RF coils and spectrometers [21].

Given these differences, evaluating the effectiveness of Haralick texture analysis in distinguishing cancerous from benign regions in prostate tissue on low-field MRI and determining whether these results align with findings from high-field MRI presents a novel and important challenge. The ability to target high-risk areas for biopsy that may be otherwise missed [22] may become a valuable biopsy planning tool for urologists.

The primary aim of this study is to assess the feasibility of using Haralick texture analysis to differentiate cancerous from normal prostate tissue on low-field, T2-weighted MRI. This work builds upon a preliminary version previously reported [23], contributing new insights into the potential of low-field MRI for prostate cancer detection.

The structure of the paper is as follows: Section 2 describes the study population and the methods used for Haralick texture analysis in 3T and low-field MRIs. The results are presented in Section 3, which is followed by the discussion in Section 4. Finally, the conclusions of the study are provided in Section 5.

## 2. Materials and Methods

### 2.1. Study Population

Patients suspected of having prostate cancer due to an elevated prostate-specific antigen (PSA) level were recruited from four clinical sites utilizing Promaxo’s system for this study. Each patient underwent multiparametric MRI (mpMRI) on a 3T MRI system without an endorectal coil. The 3T images were acquired using MRI systems from three manufacturers: Philips Medical Systems, General Electric Medical Systems, and Siemens Medical Systems, from five different models: Ingenia, Verio, DISCOVERY MR750, MAGNETOM Altea, and Signa HDxt. Suspicious ROIs were annotated on the 3T, T2-weighted images by a radiologist and assigned a PI-RADS score. Patients subsequently underwent an Institutional Review Board (IRB)-approved transperineal biopsy with the guidance of the low-field (58–74mT) MRI system (Promaxo Inc., Oakland, CA, USA). Images were acquired without endorectal probes. During the biopsy procedure, the high-field, T2-weighted MR images with annotations were co-registered with the low-field, T2-weighted images and the suspicious regions identified by the radiologist were sampled. Following histopathologic analysis, biopsied lesions with Gleason score 4+3 and higher were identified along with the corresponding, manually registered 3T and low-field, T2-weighted image slices and ROIs. In total, 21 patients with 28 lesions, all having a Gleason score of 4+3 or higher (typically associated with clinically significant prostate cancer), were included in this study.

### 2.2. Texture Analysis

**Figure 1:**
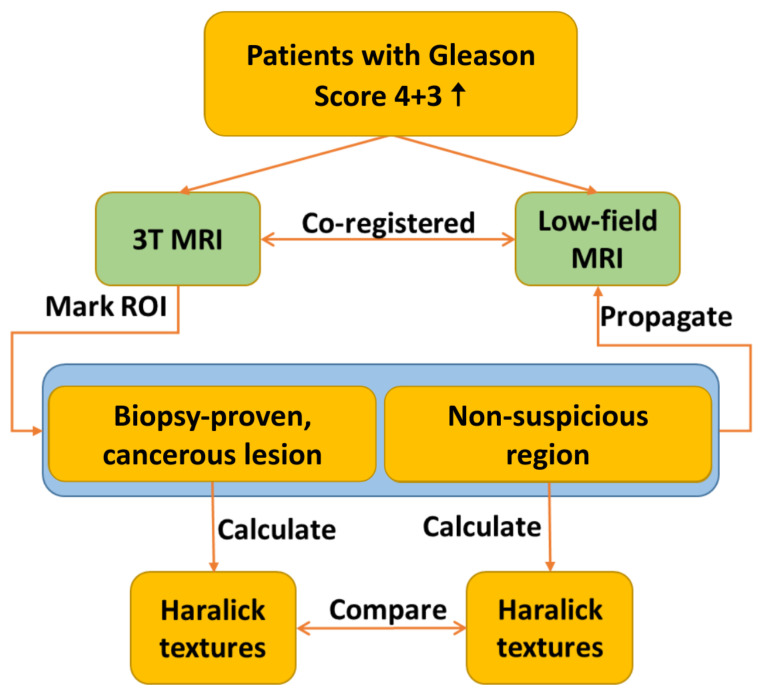
Texture analysis flowchart for both high-field and low-field MR images.

Figure 1 provides a flowchart detailing the texture analysis workflow. The process involves the registration of 3T and low-field MR images, which is followed by annotating ROIs on the co-registered images. Selected Haralick texture features are then calculated for the annotated ROIs, and the results are compared across the two imaging modalities.

#### 2.2.1. Image Registration and ROI Annotation

First, we rigidly registered 3T and low-field, T2-weighted MRI volumes to ensure that texture features were calculated from the same regions of both high-field and low-field images. This means that a rigid transformation with rotation and translation is applied to the coordinate locations of the 3T image to interpolate the low-field image onto the frame of reference of the 3T image. These transformations may be applied in succession until all image features are aligned between the two images: $I_{3T}$ and $I_{\mathrm{low}\text{-}\mathrm{field}}T$ where $T=T_{N\text{-}1}\left(T_{N\text{-}2}\left(...,T_{0}\right)\right)$, and $T_{i}\left(x\right)\;=\;\mathrm{Rx}\;+\;t$, where x represents the location in the 3T image, R is a rotation matrix and t is a translation vector. The radiologist-performed annotations were propagated from the 3T images to the co-registered, low-field images.

ROIs containing clinically significant (Gleason score 4+3 or higher) prostate cancer based on the histopathological analysis of biopsy specimens were identified. If multiple cores were collected for a single ROI with discordant results, then portions of the ROIs that were negative for prostate cancer on biopsy were excluded. For each cancerous ROI, a secondary ROI of identical size was drawn on the same slice in a non-suspicious region of the prostate, which was presumed to be normal tissue. In total, 28 image slices from 21 patients were analyzed with seven patients presenting more than one lesion, amounting to a total of 28 lesions.

For both 3T and low-field datasets, we normalized the images and rescaled them to the intensity of 64 gray levels. For each ROI, we computed the Gray Level Co-occurrence Matrix (GLCM) in four directions on transverse 2D slices.

#### 2.2.2. Haralick Features: Energy, Correlation, Contrast, and Homogeneity

A total of 14 Haralick features were introduced as texture measures to assess the relationships between pixel intensities within an image [24]. These features were derived from the GLCM, which is a two-dimensional histogram that captures the frequency of co-occurrence of two pixel intensities at a certain offset. The values in GLCM are the counts of frequencies of the neighboring pairs of image pixel values. The GLCM was typically normalized by dividing by the total number of accumulated co-occurrences. We made the GLCM symmetric for the best performance of texture calculations and for overcoming the problem of the window edge pixels. In a normalized symmetrical GLCM, the diagonal elements represent pixel pairs with no gray level difference, and the farther away from the diagonal, the greater the difference between pixel gray levels [25].

Texture measures are the various single values used to summarize the normalized symmetrical GLCM in different ways. These texture features are correlated with each other. They are divided into three groups that are independent of each other: the Contrast group that uses weights related to the distance from the GLCM diagonal, the Orderliness group that measures how often a given pair of two gray levels occur within a window, and the Descriptive Statistics group. We limit our analysis to Energy (Orderliness group), Correlation (Descriptive Statistics group), Contrast and Homogeneity (Contrast group) based on prior literature identifying them as useful for distinguishing cancer by outcomes [18].

The equations to calculate these measures are shown below.
**   Energy****   Contrast****Homogeneity****   Correlation**$$\;\sqrt{\sum_{i,j=0}^{N-1}P_{i,j}^{2}}$$$$\;\sum_{i,j=0}^{N-1}P_{i,j}{\left(i-j\right)}^{2}$$$$\;\sum_{i,j=0}^{N-1}\frac{P_{i,j}}{1+{\left(i-j\right)}^{2}}$$$$\;\sum_{i,j=0}^{N-1}P_{i,j}\frac{\left(i-\mu_{i}\right)\left(j-\mu_{j}\right)}{\sqrt{\sigma_{i}^{2}\sigma_{j}^{2}}}$$        (1)      (2)    (3)        (4)** **where $\mu_{i}=\sum_{i,j=0}^{N-1}iP_{i,j}$   (5)    and    $\sigma_{i}=\sqrt{\sum_{i,j=0}^{N-1}P_{i,j}{\left(i-\mu_{i}\right)}^{2}}$     (6)

With *i*, *j* representing the row and column number.

$P_{i,j}$ is the value for the cell *i*, *j* of the normalized GLCM.

Note that when the GLCM is symmetrical, $\mu_{i}=\mu_{j}$ and $\sigma_{i}=\sigma_{j}$.

In addition to evaluating texture features over the entire ROI, we also assessed the variation in the pixel-to-pixel relationship for different areas of the image by generating texture feature maps using a sliding window technique. For each pixel in the image, the four texture metrics were calculated for an image window and centered around the pixel, and the resulting values were assigned to that pixel position. These maps provide a visual representation of the spatial variations in the texture measures.

#### 2.2.3. Methods for Computing the Texture Features

Two methods for calculating texture features were implemented. Method 1 extracts Haralick texture measures within respective ROIs of cancerous and non-suspicious regions. Method 2 creates four texture maps which are Contrast, Energy, Correlation, and Homogeneity to evaluate the pixel-to-pixel relationship in cancerous and non-suspicious regions.

### 2.3. Statistical Analysis

A power analysis was performed to verify that the sample size was sufficient to detect meaningful differences in the four Haralick texture features between cancerous and non-suspicious regions with high confidence. The effect size was estimated separately for each texture feature in low-field images using both methods. With a power of 0.80 and a significance level at 0.05, commonly accepted thresholds in statistical analysis, the required sample size to ensure reliable discrimination between normal and abnormal tissues was calculated.

To compare the mean values of the texture features between the cancerous and non-suspicious groups, a Welch’s *t*-test was employed, which assumes unequal variances between the groups. A significance level was set to 0.05 with the null hypothesis for the test stating that there is no difference in the means of the two groups. If the calculated *p*-value for each of the four features is consistently ≤0.05, the null hypothesis is rejected, and we can conclude that the differences in all four features between the cancerous and non-suspicious groups are statistically significant.

## 3. Results

To quantify the differences in texture features between cancerous and non-suspicious regions in both 3T and low-field settings, we first calculated the four Haralick features within the respective ROIs of the images using Method 1. The four Haralick texture maps were then generated, and subsequent quantification of the texture features within the annotated ROIs was performed using Method 2. Finally, statistical and power analyses were conducted to assess the significance of the texture differences between the two tissue types.

**Figure 2:**
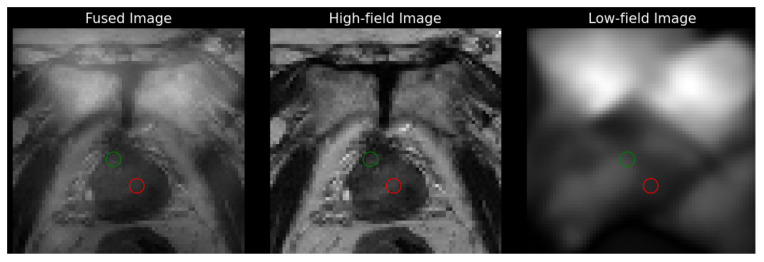
Co-registered, T2-weighted 3T and low-field MR images of a 77-year-old male with Gleason score 4+5 prostate cancer. The cancerous lesion (red) and a non-suspicious region with the same radius (green) are marked.

Figure 2 shows an example of a fused 3T, T2-weighted image registered to the low-field image (left) and a separate co-registered 3T image (middle) from low-field image (right), respectively, from a 77-year-old patient diagnosed with prostate cancer (Gleason score 4+5). The biopsy-proven cancerous lesion is marked with a red circle, and a non-suspicious region of the same size is marked with a green circle. Texture analysis is then conducted within these annotated ROIs.

**Figure 3:**
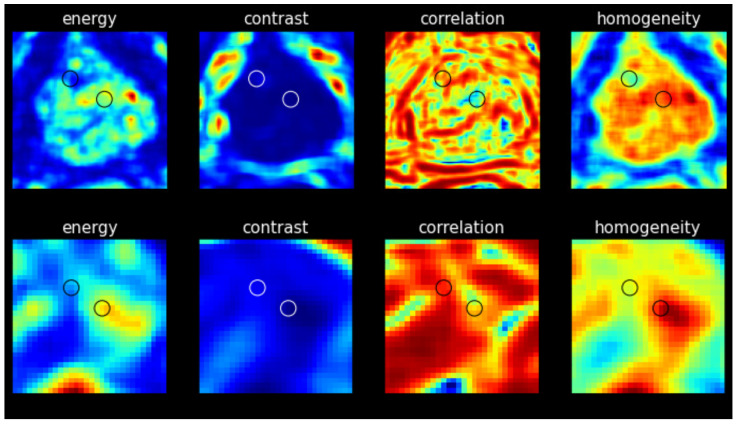
**Top**: Texture features maps on 3T image. **Bottom**: Texture features maps on low-field image. Left ROI circle: non-suspicious region. Right ROI circle: cancerous region.

Four texture maps corresponding to Haralick features were generated for each image slice (3T and low-field), with the results were obtained from the representative image of the same patient shown in Figure 3. Each map provides a visual representation of the texture measure across the image with discernible differences observed between cancerous and non-suspicious ROIs in both the 3T and low-field MR images. Note that in Figure 2 and Figure 3, the 3T image was transformed to the low-field image’s frame of reference with the same pixel spacing for better visualization.

**Figure 4:**
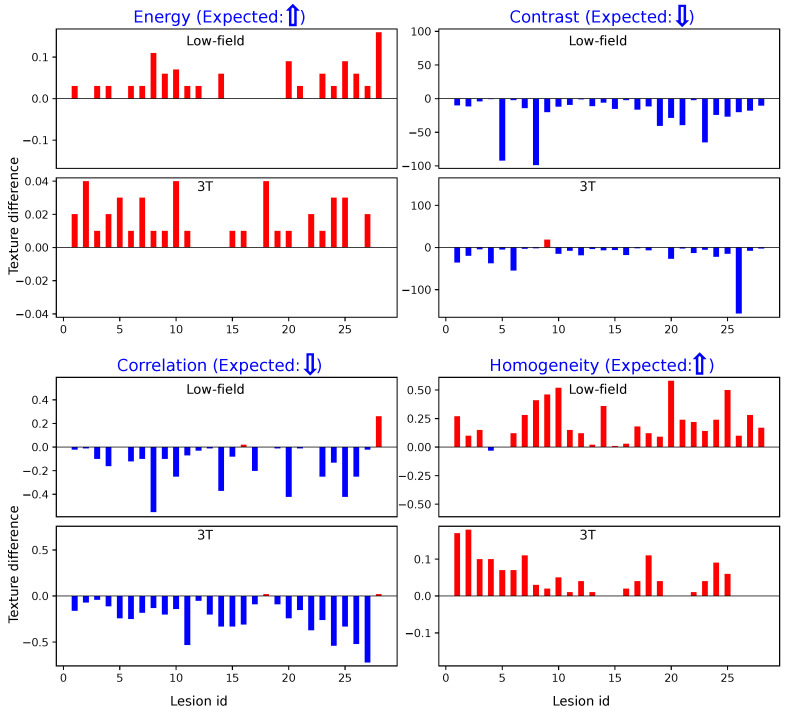
Texture measurements within ROIs using Method 1. Red indicates an increase in texture values from non-suspicious to cancerous regions, while blue represents a decrease. The expected trends in the differences between non-suspicious and cancerous regions are indicated by upward or downward arrows next to each texture title. **Top**: Differences in texture values on low-field MRI. **Bottom**: Differences in texture values on 3T MRI.

Figure 4 illustrates the differences in texture features within designated ROIs for cancerous versus non-suspicious regions in the same patient, which were derived using Method 1. Across both 3T and low-field MR images, cancerous regions consistently exhibit lower values for Contrast and Correlation, while Energy and Homogeneity values are higher. These findings align closely with prior reports on high-field (3T) imaging, as noted in the literature [18], reassuring the effectiveness of these texture-based markers in characterizing cancerous tissues.

**Figure 5:**
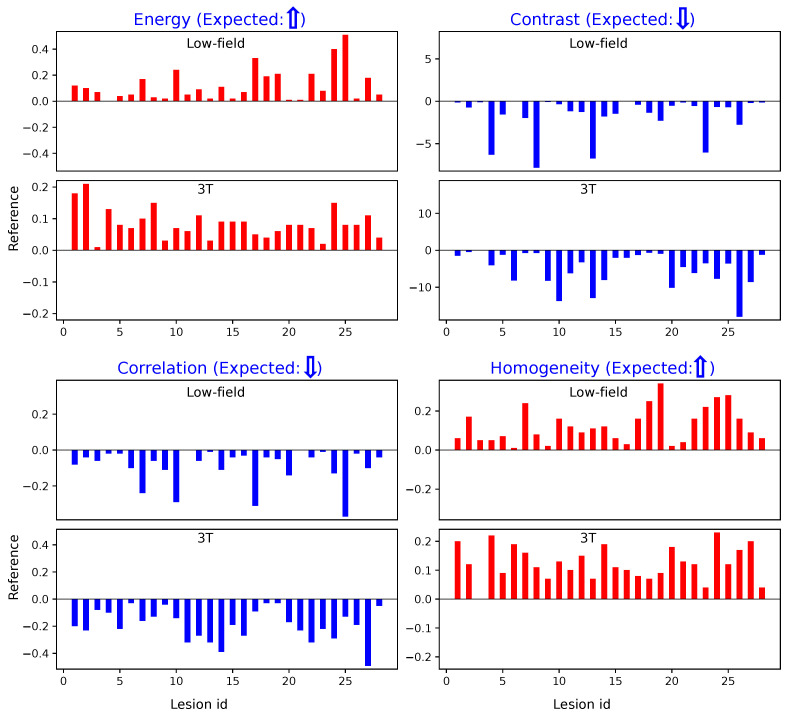
Texture measurements within ROIs using Method 2. Red indicates an increase in texture values from non-suspicious to cancerous regions, while blue represents a decrease. The expected trends in the differences between non-suspicious and cancerous regions are indicated by upward or downward arrows next to each texture title. **Top**: Differences in texture values on low-field MRI. **Bottom**: Differences in texture values on 3T MRI.

Similarly, Figure 5 illustrates the results obtained using Method 2. The trends observed are consistent with those from Method 1: cancerous regions show elevated Energy and Homogeneity values and reduced Contrast and Correlation values compared to non-suspicious regions. These parallel findings across both methods further demonstrate the robustness of our texture analysis approach.

The power analysis results indicated that Cohen’s d effect sizes for Energy, Contrast, Correlation, and Homogeneity on low-field images, calculated using both Methods 1 and 2, all exceeded 0.5 in absolute values. These medium-to-large effect sizes indicate meaningful and observable differences between normal and cancerous tissue regions, which were likely to achieve statistical significance with adequate sample sizes. Based on these effect sizes, the required sample sizes for Energy, Contrast, Correlation, and Homogeneity were determined as 20, 10, 18, and 6, respectively, using Method 1, and 13, 28, 8, and 18 using Method 2. These results confirm that our dataset size meets the analysis requirements.

Table 1 presents the *p*-values from the *t*-test assessing the relationship between the four Haralick texture features in non-suspicious versus cancerous regions in both 3T and low-field images, using Methods 1 and 2. All *p*-values are consistently below the 0.05 threshold, with most falling significantly below 0.05, indicating that the observed trends are statistically significant across samples from both low-field and 3T images.

## 4. Discussion

In this study, we evaluated the feasibility of using Haralick texture features to distinguish cancerous from non-suspicious regions in low-field, T2-weighted, axial MRI. Given the absence of prior studies in the literature applying Haralick texture analysis in low-field MRI, we began by computing Haralick texture features on high-field, T2-weighted axial images as an initial test of the methodology. Specifically, we employed Methods 1 and 2, as detailed in Section 2.2.3, for extracting and computing the Haralick texture features. The results obtained from high-field MRI closely align with those reported by Wibmer et al. [18], particularly for the same patient category, further validating our approach and its applicability to high-field imaging. Next, we tested our hypothesis regarding the capability of Haralick texture features to differentiate cancerous from non-suspicious regions in low-field MRI. We aimed to assess how well the four texture features (Contrast, Energy, Correlation, and Homogeneity) performed in low-field images in comparison to their performance in high-field MRI. By evaluating both sets of properly registered images, we sought to explore the potential of Haralick texture features to maintain their discriminatory power even in a low-field setting, where signal-to-noise ratio is typically lower and image resolution may be less optimal.

Our findings confirm that Haralick texture features derived from high-field, T2-weighted axial images are effective in differentiating cancerous from non-suspicious prostate tissues. More importantly, we discovered that the discriminatory power of these features extends successfully to low-field MRI, which suggests that Haralick texture analysis could be a reliable tool for cancer detection in lower-field systems. This result highlights the robustness of Haralick texture features and demonstrates that despite the typically lower image quality of low-field MRI, these features remain effective in distinguishing between tissue types.

Furthermore, we observed a high degree of concordance between the two methods of computing Haralick texture features on both high- and low-field MRI. This consistency in feature extraction was evident across the small cohort of patients tested in this feasibility study, which reinforced our confidence in the applicability of these methods to both high-field and low-field settings.

Despite the limited cohort, we were able to perform meaningful statistical analyses to successfully address the primary objective of this study: to assess the initial feasibility of Haralick texture analysis in differentiating cancerous from non-suspicious ROIs on low-field MRI. One limitation is that our datasets were quantified on clinically significant cancers (Gleason score of 4+3). Further analysis will need to be performed to test sensitivity to lower grade cancers. Additionally, the use of biopsy samples rather than whole-mount prostatectomy specimens for pathological validation introduces a potential source of variability, as biopsy samples may not fully represent the entire tumor or surrounding tissue. Consequently, the non-suspicious regions selected lacked corresponding pathological confirmation. These selection of non-suspicious regions were selected based on visual identification from high-field T2-weighted MRI, which, despite efforts to minimize bias, could still be influenced by the subjective nature of image interpretation. To mitigate this potential bias, we incorporated diffusion-weighted imaging (DWI) as an additional tool to support the region selection process. The predictive power of the Haralick features, as demonstrated in this study, will be further investigated as more data are collected. Larger, more diverse datasets will help validate the robustness of these features across different patient populations and enhance the generalizability of our findings. In future work, we will also explore the potential for integrating Haralick texture features with other imaging biomarkers to enhance the accuracy of prostate cancer detection in low-field MRI.

## 5. Conclusions

In this feasibility study, we explored Haralick texture features on both high- and low-field MRI to distinguish clinically suspicious prostate regions from normal tissue, specifically focusing on low-field, low-resolution T2-weighted MRI. This study is the first to extend Haralick texture analysis from high-field MRI to the more accessible and cost-effective low-field MRI, highlighting its potential to impact clinical practice. The features studied demonstrated similar trends observed in high-field images. While a larger cohort will allow a more comprehensive assessment of interpatient and cancer-grade variability, this preliminary analysis demonstrated consistent and statistically significant differences across all four Haralick features between non-suspicious and cancerous prostate regions of intermediate grade. We are currently acquiring additional images as part of an ongoing clinical study to validate these findings in a broader patient population across different grades of prostate cancer. This study has shown the potential of using Haralick texture features to differentiate cancerous tissues in low-field MRI, and these features could also help guide biopsy planning to ensure high-risk areas of the prostate that may otherwise be missed are appropriately sampled.

## 6. Patents

The patent resulting from the work reported in this manuscript has been assigned with the International Publication Number WO2024025828A1.

## Figures and Tables

**Table 1:** *p*-values for the four Haralick features computed for low-field and 3T images using both Methods 1 and 2.

	Method 1		Method 2	
	**Low-Field**	**3T**	**Low-Field**	**3T**
Energy	***p*** **= 0.0005**	*p* = 0.0125	***p*** **= 0.0011**	*p* = $3\times 10^{-5}$
Contrast	***p*** **= 0.0002**	*p* = 0.0449	***p*** **= 0.0285**	*p* = 0.0017
Correlation	***p*** **= 0.0057**	*p* = $1\times 10^{-5}$	***p*** **= 0.0001**	*p* = $1\times 10^{-8}$
Homogeneity	***p*** **= $8\times 10^{-6}$**	*p* = 0.0026	***p*** **= 0.0038**	*p* = 0.0003

## Data Availability

The datasets presented in this article are not readily available due to data privacy restrictions.

## Abbreviations

The following abbreviations are used in this manuscript:

MRI	Magnetic Resonance Image
ROI	regions of interest
IRB	Institutional Review Board
PI-RADS	Prostate Imaging–Reporting and Data System
GLCM	Gray Level Co-occurrence Matrix
ADC	Apparent Diffusion Coefficient
